# Natural Occurrence of Entomopathogenic Fungi as Endophytes of Sugarcane (*Saccharum officinarum*) and in Soil of Sugarcane Fields

**DOI:** 10.3390/insects12020160

**Published:** 2021-02-13

**Authors:** Trust Kasambala Donga, Richard Meadow, Nicolai V. Meyling, Ingeborg Klingen

**Affiliations:** 1 Bunda College Campus, Crop and Soil Sciences Department, Lilongwe University of Agriculture and Natural Resources (LUANAR), P.O. Box 219 Lilongwe, Malawi; 2 Department of Plant Sciences, Faculty of Biosciences, Norwegian University of Life Sciences (NMBU), P.O. Box 5003, 1432 Ås, Norway; richard.meadow@nmbu.no; 3 Norwegian Institute of Bioeconomy Research (NIBIO), Biotechnology and Plant Health Division, Høg-skoleveien 7, 1430 Ås, Norway; nvm@plen.ku.dk (N.V.M.); ingeborg.klingen@nibio.no (I.K.)

**Keywords:** entomopathogenic fungi, *Beauveria bassiana*, Malawi

## Abstract

**Simple Summary:**

Sugarcane, an important cash crop in Malawi, is susceptible to numerous insect pests, and many farmers rely heavily on chemical insecticides for their control. Biopesticides containing insect pathogens are used in several countries outside Malawi; however, the occurrence and use of insect pathogens is limited in Malawi. In this study, we evaluated the natural occurrence of insect pathogenic fungi in sugarcane (*Saccharum officinarum*) and in soil samples from sugarcane fields in Chikwawa District, southern Malawi. Insect pathogenic fungi from soil were isolated by baiting using larvae of the greater wax moth (*Galleria mellonella*). Insect pathogenic fungi were also isolated from surface-sterilized sugarcane leaves, stems, and roots. We found three types of insect pathogenic fungi: *Beauveria bassiana*, *Metarhizium* spp., and *Isaria* spp. *Beauveria bassiana* and *Isaria* spp. were found mostly from sugarcane leaves and stems, while *Metarhizium* spp. was mainly found in soils. To the best of our knowledge, this is the first report of *B. bassiana* and *Isaria* spp. occurring naturally as endophytes in sugarcane. Further, it is the first report of *B. bassiana*, *Isaria* spp. and *Metarhizium* spp. in the soil of sugarcane fields in Africa.

**Abstract:**

The natural occurrence of entomopathogenic fungal endophytes in sugarcane (*Saccharum officinarum*) and in soil samples from sugarcane fields was evaluated in Chikwawa District, southern Malawi. Fungi from soil were isolated by baiting using *Galleria mellonella* larva. Fungal endophytes were isolated from surface-sterilized plant tissue sections. Forty-seven isolates resembled the genus *Beauveria*, 9 isolates were *Metarhizium*, and 20 isolates were *Isaria*. There was no significant difference in the number and type of fungal isolates collected from soil and from plant tissue. There was, however, a significant difference in the part of the plant where fungal species were isolated, which fungal species were isolated, and the number of fungal species isolated at each location. Phylogenetic analysis of 47 *Beauveria* isolates based on DNA sequencing of the Bloc intergenic region indicated that these isolates all belonged to *B. bassiana* and aligned with sequences of *B. bassiana* isolates of African and Neotropical origin. The Malawian *B. bassiana* isolates formed a distinct clade. No larvae died from infestation by multiple fungi. To the best of our knowledge, this is the first report of *B. bassiana* and *Isaria* spp. occurring naturally as endophytes in sugarcane. Further, it is the first report of *B. bassiana*, *Isaria* spp., and *Metarhizium* spp. in the soil of sugarcane fields in Africa.

## 1. Introduction

Entomopathogenic fungi (EPF) in the order Hypocreales are known to infect and kill arthropods, occur in the soil environment, and interact with plants as endophytes [1,2,3,4,5,6]. Depending on their biology and ability to grow on artificial media, these fungi may be used in biocontrol of crop pests [6,7]. Entomopathogenic fungi can cause epizootics in populations of soil-dwelling insects and mites [8], and fungal isolates of the genera *Beauveria* and *Isaria* (Hypocreales: Cordycipitaceae) and *Metarhizium* (Hyporeales: Clavicipitaceae) are developed for inoculative or inundation biological control of agricultural pests [9,10,11,12,13].

The natural occurrence and diversity of entomopathogenic fungi in terrestrial ecosystems may be affected by many factors such as climate, habitat, soil properties, plant species, agricultural practices, and sampling method [10,13,14,15,16]. Klingen and Haukeland [15] suggest that the use of chemical pesticides, especially fungicides but also herbicides, may reduce the prevalence of entomopathogenic fungi in the soil. Further, Klingen et al. [13] isolated entomopathogenic fungi more frequently in soil samples from organic than from conventional arable fields. Likewise, Clifton et al. [5] reported that soils from organic soybean or maize fields had more entomopathogenic fungi than corresponding conventional fields and that the occurrence of entomopathogenic fungi was negatively affected by tillage, elevated nitrogen content of soil, and herbicide and fungicide application. Furthermore, Ramos et al. [17] recovered *Beauveria bassiana* more frequently in soil samples and root tissues from organic than conventional bean fields. In a review of the literature, Meyling and Eilenberg [10] reported that *Metarhizium anisopliae* sensu lato generally was most prevalent in soils collected from agricultural fields, compared to undisturbed areas such as hedgerows, while *B. bassiana* was more frequently isolated from soils from the undisturbed areas. Besides occurrence in soils, *Beauveria* spp. and *Metarhizium* spp. have been isolated as endophytes from perennial woody plants such as coffee, pine, and cocoa [1,17,18,19,20] and non-woody plants such as beans and maize [17,21,22]. 

Although *B. bassiana* and *M. anisopliae* are known to be effective against insect pests that infest sugarcane [20,23,24,25,26], few studies have focused on natural occurrence of entomopathogenic fungi in sugarcane cropping systems in Africa [24,27]. Ngubane et al. [27] isolated *Metarhizium anisopliae*, *Beauveria bassiana*, *Beauveria brongniartii*, and *Lecanicillium lecanii* from various insect cadavers collected from sugarcane fields in six countries in southern Africa. Goble et al. [24] characterized *Beauveria* spp. isolates obtained from insect, soil, and root origin from sugarcane production sites in South Africa. However, there are no published studies on the natural occurrence of entomopathogenic fungi from the Hypocreales as endophytes in sugarcane of sugarcane cropping systems from Africa. The aims of the present study were therefore to investigate whether these entomopathogenic fungi occur naturally in soils and in sugarcane tissues collected in sugarcane fields in Malawi and whether the prevalence of the fungi varies among sugarcane cropping systems.

## 2. Materials and Methods 

### 2.1. Description of Sugarcane Production, Location, and Sampling of Plants and Soil

Sugarcane is vegetatively cultivated using short-stem cuttings (referred to as plant cane) and from old growth (referred to as ratoon cane). In many African countries, smallholder farmers grow sugarcane for home consumption but also sell raw sugarcane in local markets [28]. These farmers grow traditional cultivars or a mixture of cultivars and intercrop the sugarcane rows with other crops such as maize and vegetables. Sugarcane is grown in seasonal wetlands of valley bottoms called “dambo” and low-lying areas called “dimba”, both of which are referred to here as “traditional” fields. Traditional farmers use a hoe for tilling the soil two or more times per year, irrigate the field as required, and apply insecticides without consideration to economic thresholds [29,30]. Commercial estates owned by foreign multinational companies also grow sugarcane for processing into sugar and other sugarcane-based products. These estates use irrigation and other cultivars that originate from both within and outside Africa. A third category of sugarcane farmers are referred to as “outgrowers”, and they grow sugarcane using the same varieties as the commercial estates either under rain-fed conditions or irrigation. Outgrowers are supposed to follow production guidelines used in commercial estates and may belong to a farmer association that provides input packages (seed, fertilizer, and herbicides) on credit or may act independently [29]. Commercial estates and outgrowers’ sugarcane fields are ploughed on average every 3.8 years. On commercial estates and at outgrowers, chemical insecticides and herbicides are applied according to economic threshold levels [29] provided by ILLOVO Malawi agronomists based at Nchalo Estate. Sugarcane is harvested green in traditional fields but is burned prior to harvesting in commercial estates and outgrowers’ fields. 

**Figure 1:**
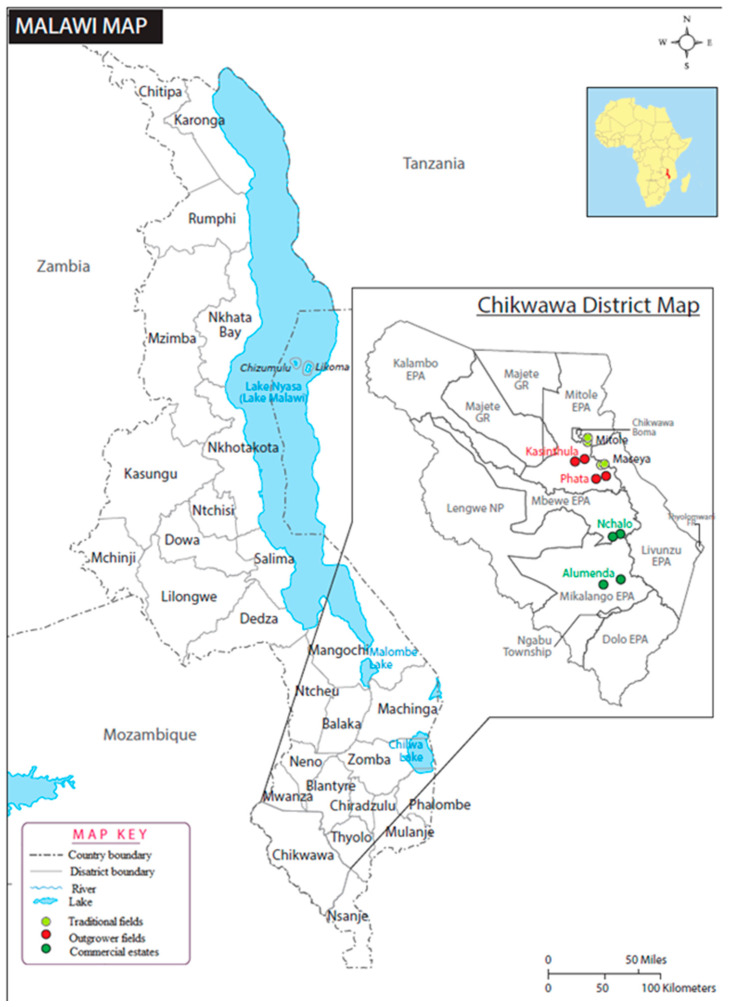
Locations of the 12 sugarcane fields sampled in Chikwawa District, southern Malawi.

In this study, field surveys were conducted from July to December 2016 in 6 locations, namely, Mitole, Maseya, Phata, Kasinthula, and Alumenda in the Chikwawa District of southern Malawi (Figure 1; Table S1). There are 3 soil associations that dominated the study area: well drained youthful soils (Cambisols, soil association A), which constitute 59%; black cracking clays (Vertisols, soil association H), comprising 22%; and Duplex soils (Luvisols, soil association C), comprising 6% [31]. The study area lies below 150 m. The climate is tropical continental with 2 seasons: the rainy season from November to April and the dry season from May to October. However, from May to July, it is relatively cool. The coldest months are June and July. The highest temperatures occur at the end of October or early November. The mean annual minimum and maximum temperatures range from 15 to 36 degrees Celsius (°C) [31]. Within each location, 2 sites were randomly selected, and a 30 × 30 m quadrat was established as a frame for 5 sampling units. In Malawi, sugarcane fields are ploughed down after an average of 3.8 harvests. Sugarcane is harvested after growing for 12–15 months. Sugarcane fields harvested 4 to 7 times, i.e., 45.6- to 105-month-old ratoon cane were sampled. The plants were sampled less than 5 months after harvest. Plants were sampled by carefully uprooting 1 plant from the center and from the 4 corners of the 30 × 30 m quadrat at each of the 2 sites per location. Collected plants (*n* = 60 in total) were placed in polyethylene bags and transported fresh and intact in 40 L cooler boxes to the laboratory for assessment within 24 h. Within each quadrate of the 12 sites, we collected 5 soil samples at a distance of 60 cm from the base of the collected plant and down to a 15 cm depth by the use of a garden spade. The spade was sterilized in 70% alcohol between samplings to prevent cross-contamination. Soil samples were then placed separately in 1 L polyethylene bags and transported immediately in 40 L cooler boxes to the laboratory for processing.

### 2.2. Isolation of Fungi

#### 2.2.1. Isolation of Endophytic Fungi from Plant Samples

Upon arrival at the laboratory, the soil was carefully shaken off the plant roots and the roots were washed with tap water. From each sampled sugarcane, 10 cm sections of stem, leaf, and root, were cut out and surface-sterilized by immersion for 2 min in 3% sodium hypochlorite followed by 2 min in 70% ethanol and then rinsed thrice for 30 s in sterile distilled water, as described by Parsa et al. [32]. Effectiveness of the sterilization process was evaluated by plating 100 µL of the last rinse water on Sabouraud dextrose agar (SDA, Oxoid) with 1% antibiotics (0.2 g penicillin, 0.2 g chloramphenicol, and 0.2 g tetracycline dissolved in 10 mL sterile distilled water, followed by filter sterilization through a 0.2 mm filter). The absence of fungal growth from the last rinse of water indicated that sterilization was successful. The sterilized plant tissue sections were dried on sterile paper for 1 min and the edges were trimmed so that the sections measured 60 mm long. The 60 mm trimmed sections were further dissected into 5 equal size pieces and all 5 pieces were pressed onto SDA in the same Petri dish. After sealing with Parafilm, the Petri dishes were incubated in the dark for 14–21 days at 25 ± 5 °C. Fungal growth emerging from the plant tissue was reisolated onto new SDA plates to obtain pure cultures. Mycelia and conidia from pure cultures were stored on silica gel at 25 ± 5 °C and later used for morphological and molecular characterization.

#### 2.2.2. Isolation of Fungi from Soil Samples

In the laboratory, the 5 soil samples per site were thoroughly mixed to produce 12 composite pooled soil samples. Soils were kept at 4 °C until processing but never for longer than 5 days. All soil samples were sieved through a 2 mm mesh sieve to remove debris. Dry soil samples were slightly moistened with sterile water while wet soils were first air-dried to remove excess water and reduce the incidence of nematodes. The widely and best-known method for selecting entomopathogenic fungi, the *Galleria mellonella* bait method described by Zimmermann [33], was used to isolate entomopathogenic fungi from soil samples. Before being used as baits, 4–5-week-old *G. mellonella* larvae were heat-conditioned as described by Woodring and Kaya [34] by immersion in 56 °C sterile water for 15 s, followed by pouring cold water on top of the larvae for 30 s and then letting the larvae rest for 1 h to recover. This was done to reduce the ability of the larvae to produce webbing while in the soil. Five live heat-conditioned *G. mellonella* were then added to a 350 mL plastic container with an aerated lid containing 300 g of the sifted soil sample and incubated for 14 days in the dark at 25 ± 5 °C. The plastic containers were inverted once every 2 days to promote larval movement through the soil. 

Containers with soil samples were checked daily, and dead larvae were removed, surface sterilized by immersing them in 70% alcohol for 10 s, rinsed thrice in sterile water for 10 s, and left to dry on a sterile paper towel. They were then individually placed in a moist chamber and incubated for 14 days at 25 ± 5 °C. Dead larvae were observed every 2 days for fungal growth, and mycelia were isolated by placing them on SDA with 0.1% antibiotics and incubated as described above. The number of dead larvae exhibiting mycosis was recorded. Fungal growth obtained from each mycosed larva was considered an isolate. Fungal isolates were stored in silica gel until morphological and molecular characterization.

### 2.3. Morphological Identification of Fungi

Entomopathogenic fungi in the Hypocreales were identified morphologically to genus level according to Humber [35] by examining under a 400× phase contrast microscope. 

### 2.4. Molecular Identification of Fungi to Species Level

The identification of entomopathogenic fungi in the Hypocreales to the species level requires molecular techniques [36,37]. Molecular analysis of fungi to species level in this study have thus far only been conducted on the 47 isolates that were morphologically identified to be in the genus *Beauveria*. 

#### 2.4.1. DNA Extraction, PCR Amplification, and Sequence Analysis

DNA extraction and PCR reactions were performed at NIBIO, Ås, Norway. DNA was extracted from *Beauveria* isolates only because *Beauveria* isolates are widely used in biological control programs and are effective against a wide range of arthropod pests that occur in sugarcane [9,10,11,12,13]. A few silica gel crystals from the stored fungal isolates were placed onto SDA plates (9 cm diameter) and incubated in the dark for 14 days at room temperature (21–25 °C) in the laboratory at NIBIO. Mycelia and conidia were then harvested by scraping off a small portion of the fungus using a sterile scalpel. The harvested mycelia and conidia were then ground to a fine powder using a mortar and pestle in liquid nitrogen before extracting the genomic DNA using a DNeasy Plant Mini kit (Qiagen, Germany) in accordance with the manufacturer’s instructions.

PCR amplification targeting the intergenic Bloc region for 47 *Beauveria* cycler was carried out using a Bio-Rad T100 Thermal. Amplification of the Bloc gene region was achieved with the primer pair B22U (5′-AGATTCGCAACGTCAACTT-3′) and B822L (5′-GTCGCAGCCAGAGCAACT-3′) [36]. PCR targeting the Bloc regions was performed. The reaction volume of 50 µL contained 1.5 µL Mm MgCL2, 1× PCR buffer, 4 µ 200 µM deoxynucleotides (dNTPs), 1 µL of each primer (10 µM), 0.1 µL 0.5U Platinum Taq DNA polymerase, and 3 µL genomic DNA. Cycling conditions for Bloc gene regions were as follows: 5 min at 95 °C denaturation followed by a touch-down protocol with 30 s denaturation at 95 °C, 30 s at 70–60 °C (reducing annealing temperature by 1 °C per cycle), and 1 min at 72 °C. An additional 30 cycles were performed including 30 s at 95 °C, 30 s annealing at 60 °C, and 1 min at 72 °C, followed by a final extension of 5 min at 72 °C.

The PCR products were visualized by gel electrophoresis—1.0% agarose gel with TBE (45 mM Tris base, 45 mM boric acid, 1 mM Ethylenediaminetetraacetic acid (EDTA) (pH 8.0)). Staining of bands with ethidium bromide (Thermo Fisher Scientific, New York, NY, USA) was performed to help with visualization of the amplified DNA through a GelDoc EQ gel imaging system equipped with PDQuest 2-D analysis software (Bio-Rad Laboratories, Califonia, USA). The size of the PCR products was determined through comparison to a 100 bp DNA ladder (New England Biolabs, Hertfordshire, UK). PCR products were diluted (where necessary) in nuclease-free water to acquire the right concentration (10–50 ng-µL) recommended for sequencing. PCR products were sent to GATC Biotech (Germany) for sequencing. Sanger sequencing was performed by GATC Biotech (Germany) using the B22U/B822L primer pair. 

#### 2.4.2. Phylogenetic Analysis

The sequences obtained from the Bloc gene analysis were traced, edited, and assembled using CLC Main workbench 7. Nucleotide sequence consensus sequences were aligned using ClustalW in BioEdit 7.2.5 [38,39]. Published sequences for *Beauveria* [24,26,36] were included in the phylogenetic analysis. Intraspecific divergence was calculated using Mega6 [40]. Preliminary neighbor-joining (NJ) and maximum likelihood (ML) trees were generated for the aligned sequences using Mega6 [40]. Both NJ and ML trees were based on the Kimura 2-parameter model K2P [40]. Using the model selection option in Mega6, we found that the Kimura 2-parameter with discreet Gamma distribution (K2 + G) was the best-fit model for our dataset on the basis of the lowest Bayesian information criterion (BIC) value. We used the best-fit model to generate ML analysis using 1000 bootstrap replications. A reference sequence of *Beauveria malawiensis* was included to root the phylogenetic tree. 

### 2.5. Data Analysis

Preliminary data exploration indicated that the data (frequencies of occurrence of soil and plant samples positive for *Beauveria* spp., *Isaria* spp., and *Metarhizium* spp. collected from soil and sugarcane plants) did not follow a normal distribution. Hence, frequency data were analyzed using non-parametric tests, one-sample option. Chi-squared (Χ^2^) tests and cross-tabulations were also computed to find relationships between location, farm type, and occurrence of EPF. Pairwise comparison test was carried out to compare the distribution of EPF across farm type. All statistical analyses were carried out in SPSS version 22 (IBM Statistics Software, IBM, New York, NY, USA).

## 3. Results

### 3.1. Morphological Identification and Frequency of Occurrence

A total of 60 sugarcane plant samples and 60 *G. mellonella* larvae were used to bait the 12 soil samples evaluated in this study. On the basis of morphological features, we identified 76 fungal isolates. Of these, 47 isolates resembled *Beauveria*, 9 isolates were *Metarhizium*, and 20 isolates were *Isaria* (Table 1). Location had a significant effect on the type of fungal species isolated (Χ² = 19.462, *d.f*. = 11, *p* = 0.035) (Table 1). Pairwise comparison indicated that distribution of EPF was the same across the category of farm type (*p* = 0.111). *Beauveria* spp. was present at all the locations sampled. There were significant differences in the part of a plant part where fungal species were isolated (Χ² = 18.750, *d.f*. = 2, *p* < 0.001), which fungal species were isolated (Χ² = 14.683, *d.f*. = 2, *p* = 0.001), and the number of fungal species isolated (Χ² = 18.750, *d.f.* = 2, *p* < 0.001). More *Beauveria* spp. were recovered from leaf tissue than in stem and root tissue.

### 3.2. Phylogenetic Analysis

**Figure 2:**
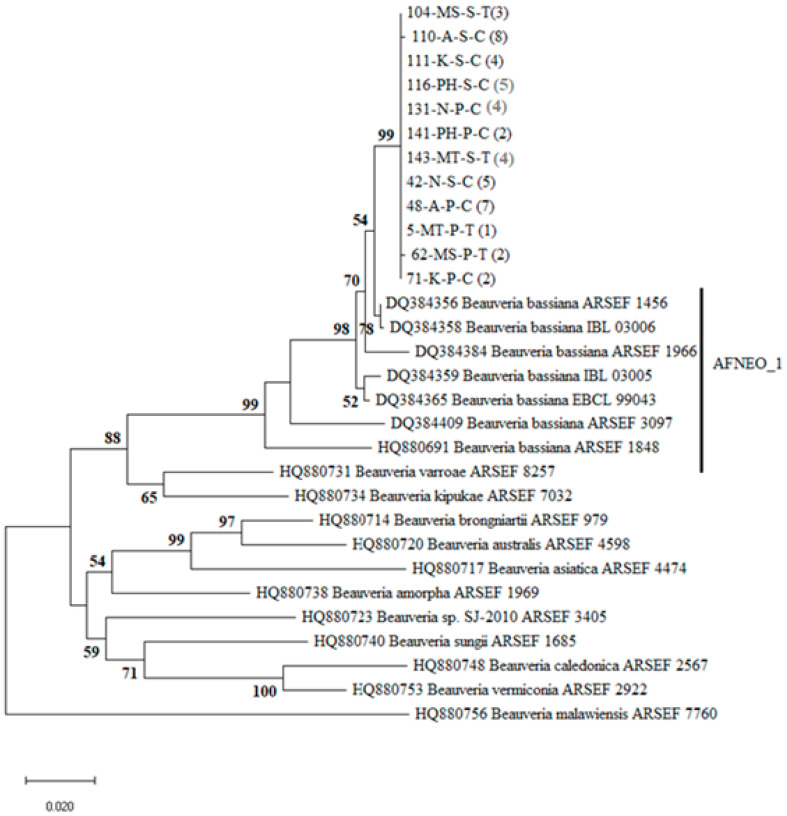
Phylogenetic tree of *Beauveria* indicating the position of Malawian isolates collected from sugarcane fields within the worldwide *Beauveria* genetic structure. The tree was inferred by the maximum likelihood (ML) method based on the Kimura 2-parameter model with a discrete Gamma distribution (K2 + G) using the Kimura 2-parameter method of intergenic Bloc region of 47 Malawian and 18 reference sequences from GenBank (given with their associated accession number). For the isolates from Malawi, the code after the isolate number denotes substrate: S = soil, P = plant; location: MS = Maseya, MT = Mitole, N = Nchalo, P = Phata, K = Kasinthula, A = Alumenda; in brackets = number of isolates. Branch support was measured through 1000 bootstrap repetitions.

Sequences for the 47 *Beauveria* isolates were generated. A highly similar sequences match search done using Basic Local Alignment Search Tool (BLAST) on the National Center for Biotechnology Information (NCBI) database indicated that the *Beauveria* isolates were *B. bassiana.* The additional 18 bloc sequences of *Beauveria* were downloaded from the GenBank for phylogenetic placement of the sequences. The alignment contained 845 positions. After eliminating gaps and missing data, we resulted in 727 nucleotide positions included in the final dataset. ML analysis based on the Bloc produced trees with similar topologies with well-resolved clusters representing isolates of five different species of *Beauveria* (Figure 2). All the 47 *Beauveria* isolates sequenced in this study had similar sequences and formed their own unique clade with high branch support, which clustered within *B. bassiana* clade (Figure 2), specifically the clade containing reference sequences from Africa (Cameroon, Côte d’Ivoire, Kenya, Togo) and the Neotropics (Brazil, Colombia, Costa Rica, Mexico, Nicaragua) denoted as AFNEO_1 by Rehner et al. [36].

## 4. Discussion

To the best of our knowledge, this is the first report of *B. bassiana* and *Isaria* spp. occurring naturally as endophytes in sugarcane. *B. bassiana* and *Isaria* spp. isolated from sterilized sugarcane plant tissue represent naturally colonized plant parts, which can have been inoculated from fungal propagules in the soil or from infected insect hosts. This expected route of entry is supported by a recent study demonstrating the ability of *B. bassiana* to experimentally establish as an endophyte of sugarcane [41]. The incidence of *B. bassiana* in leaf tissue in our study could also have been a result of aerial deposition of *B. bassiana* propagules [10,42]. In addition, insects have the ability to transport *B. bassiana* to plant surfaces [43]. As an endophyte, *B. bassiana* has been isolated from plant tissues of common bean [17], coffee and cocoa plants [1,11,19], faba beans [44], maize [21], and pine needles [18]. *Isaria* spp. (formerly *Paecilomyces*) [32] have been reported as endophytes in rice (*Paecilomyces* sp.) [45], mangrove (*Paecilomyces varioti*) [46], banana (*Paecilomyces* sp.) [9], and coffee plants (*Paecilomyces* cf. *fumosoroseus*, *P*. cf. *javanicus*) [47]. Endophytism between entomopathogenic fungi such as *B. bassiana* and *Metarhizium* (not all *Isaria* spp. are pathogenic to insects) and plants is considered to be detrimental to insect pests [48,49,50]. The negative impact on insect pests may be direct by infection, induction of secondary metabolites (terpenoids) involved in plant defense against herbivory, or synthesis of herbivore-induced plant volatiles (HIPVs) and kairomones that can be used by parasitoids in locating their insect hosts [9,24,48,49,50].

This is the first report of the natural occurrence of *Beauveria bassiana* in soil from sugarcane fields in Malawi. The frequency of occurrence of *Beauveria* spp. differed among locations. Several previous studies indicate that convectional agricultural practices such as application of synthetic pesticides decrease the natural incidence and diversity of the Hypocreales [14,16,17,51]. However, farm type did not significantly affect the distribution of EPF across locations. Goble et al. [52] and Meyling et al. [53] also found no difference in the occurrence of *B. bassiana* and *M. anisopliae* in organically and conventionally managed citrus orchards in South Africa and white cabbage and onion fields in Denmark, respectively. On, the contrary, in Cuba, the overall frequency of occurrence of *Beauveria* isolates in conventional bean fields was significantly lower than that in organic fields [17]. All the sugarcane fields sampled were subjected to conventional tillage, i.e., inverting and loosening of soil after harvest. This practice is more frequent in traditional fields (multiple times in a year) compared to commercial estates and outgrowers’ fields (performed once every 3.8 years). Some studies have negatively associated abundance of entomopathogenic fungi in agricultural fields with conventional tillage [5,21,54]. In the sugarcane fields studied, insecticides were applied [29]. Insecticides reduce insect populations in a field and possibly the endophytic inoculum in the plant due to fewer fungal-infected hosts. This may be important for the dissemination of entomopathogenic fungal inoculum between the soil and the phyllosphere [14,55,56,57,58]. However, several other factors that are known to influence the natural occurrence and diversity of entomopathogenic fungi in agroecosystems could also have an impact on the present results [5,14,15,16,59,60,61,62], although these factors were not considered in the present study. 

Our phylogenetic analysis placed all the 47 Malawian *Beauveria* spp. isolates within the same *B. bassiana* clade, which is closely related to *B. bassiana* of other countries in Africa (Cameroon, Côte d’Ivoire, Kenya, and Togo) and Neotropical regions (Brazil, Colombia, Costa Rica, Mexico, and Nicaragua) referred to as AFNEO_1 [36]. The close relationship among the *B. bassiana* isolates from the sugarcane fields characterized in the present study indicate that they all represent a single *B. bassiana* population distributed among the six locations in Chikwawa district in southern Malawi. Our results are similar to those of Ramos et al. [17], who found limited diversity among *B. bassiana* isolates from common bean fields in Cuba distributed in samples of soil and plant material. These two *B. bassiana* population studies are in contrast to reports from Europe, where two separate clades of *B. bassiana* were found infecting pollen beetles in Switzerland [59]. In another study conducted in Denmark, several clades of *B. bassiana* coexisted in the soil of the hedgerow bordering an agriculture field [60]. The reason for the seemingly limited diversity within *B. bassiana* populations from agricultural systems in East Africa and the Caribbean compared to Europe could be related to the tropical climate or limited dispersal mechanisms within these regions. It has previously been reported that *B. bassiana* is effective when applied against arthropod pests that infest sugarcane [20,23,24,25] and the naturally occurring fungi may contribute to the regulation of pest populations [63,64,65,66,67].

## 5. Conclusions

The present study is the first to report of *B. bassiana* and *Isaria* spp. as naturally occurring endophytic fungi in sugarcane. Further, the results suggest that *B. bassiana* and *Isaria* spp. constitute a naturally occurring reservoir of entomopathogenic fungi in soils and crop tissues of conventionally and traditionally grown sugarcane. The limited molecular diversity among the *B. bassiana* isolates in Malawi suggests that they comprise a single population with low gene flow and with the ability to both infect insects and colonize plant tissues. Future studies should focus on determining the effect of *B. bassiana* as endophytes of sugarcane against insect pest populations. 

## Figures and Tables

**Table 1:** Number of *Beauveria* spp., *Metarhizium* spp., and *Isaria* spp. obtained from soils (*n* = 60) and as endophytes of sugarcane (*Saccharum officinarum*) tissues (root, *n* = 60; stem, *n* = 60; leaf, *n* = 60) from two commercial estates (Alumenda and Nchalo), two outgrowers fields (Kasinthula and Phata), and two traditional fields (Maseya and Mitole) in the Chikwawa District, southern Malawi.

Location	Fungal Species Isolated						Total
	*Beauveria* spp.		*Metarhizium* spp.		*Isaria* spp.		
	Plant	Soil	Plant	Soil	Plant	Soil	
Alumenda	7	8	0	3	3	0	21
Nchalo	4	5	0	0	3	1	13
Kasinthula	2	4	0	0	4	2	12
Phata	2	5	0	0	2	2	11
Maseya	2	3	0	1	0	0	6
Mitole	1	4	1	4	3	0	13
Total	18	29	1	8	15	5	76

## Data Availability

Sequences for the Malawian *Beauveria bassiana* are available on NCBI GenBank. GenBank accession numbers MW570829-MW570841.

## Supplementary Materials

The following are available online at https://www.mdpi.com/2075-4450/12/2/160/s1: Table S1. Study site description.
