# Supplementary material for: Natural Occurrence of Entomopathogenic Fungi as Endophytes of Sugarcane (Saccharum officinarum) and in Soil of Sugarcane Fields
Source: Insects. 2021 Feb 13;12(2):160. doi: 10.3390/insects12020160 (PMC7917985; doi:10.3390/insects12020160)
Supplement: Supplementary file 1 [file insects-12-00160-s001.pdf]

Table S1: Study sites description

Location	Field type	Site #	GPS Coordinates	Date sampled	Crops grown	Cultivar grown	Pesticides used	Tillage system
Alumenda	Commercial	Site 1<br>Site 2	16°22'37.7"S 34°54'25.5"E<br>16°23'24.6"S 34°51'45.2"E	7 Oct 2016	sugarcane	MN1	Acetamiprid, (acetamiprid 200g/L)	Conventional using tractor ploughs
Nchalo	Commercial	Site 3<br>Site 4	16°16'17.2"S 34°53'09.8"E<br>16°15'52.6"S 34°54'19.8"E	15 Oct 2016	Sugarcane	MN1	Acetamiprid, (acetamiprid 200g/L)	Conventional using tractor ploughs
Kasinthula	Outgrower	Site 5<br>Site 6	16°04'44.6"S 34°48'46.4"E<br>16°05'07.6"S 34°47'39.9"E	22 Dec 2016	Sugarcane	MN1, N32	Cypermethrin 200 EC (200g/L cypermethrin)<br>Acetamiprid, (acetamiprid 200g/L)	Conventional using tractor ploughs
Phata	Outgrower	Site 7<br>Site 8	16°07'18.9"S 34°51'56.7"E<br>16°07'30.3"S 34°51'03.6"E	21 Dec 2016	Sugarcane	N32	No information	Conventional using tractor ploughs
Maseya	Traditional	Site 9<br>Site 10	16°05'36.9"S 34°51'18.5"E<br>16°05'37.2"S 34°51'18.0"E	8 Oct 2016	Sugarcane, maize, onions, tomato.	Mixture of local cultivars such as Msenjere, Chiutsa and Mkono wa Mwana	Dimethiote 40EC (400g/L dimethoate)<br>Cypermethrin 200 EC(200g/L cypermethrin)<br>Aceta 20 SL (200g/L acetamiprid)<br>Marshal 250 EC (25% v/v carbosulfan)	Conventional using hoes
Mitole	Traditional	Site 11<br>Site 12	16°02'12.5"S 34°49'12.5"E<br>16°02'11.9"S 34°49'08.2"E	16 Oct 2016	Sugarcane, maize, onions, tomato	Mixture of local cultivars	Dimethiote 40EC (400g/L dimethoate)	Conventional using hoes

						such as Msenjere, Chiutsa and Mkono wa Mwana	Aceta 20 SL (200g/L acetamiprid)<br>Cypermethrin 200 EC(200g/L cypermethrin)<br>Marshal 250 EC (25% v/v carbosulfan)	
